# Supplementary figures and images for: Measles virus exits human airway epithelia within dislodged metabolically active infectious centers
Source: PLoS Pathog. 2021 Aug 12;17(8):e1009458. doi: 10.1371/journal.ppat.1009458 (PMC8384213; doi:10.1371/journal.ppat.1009458)

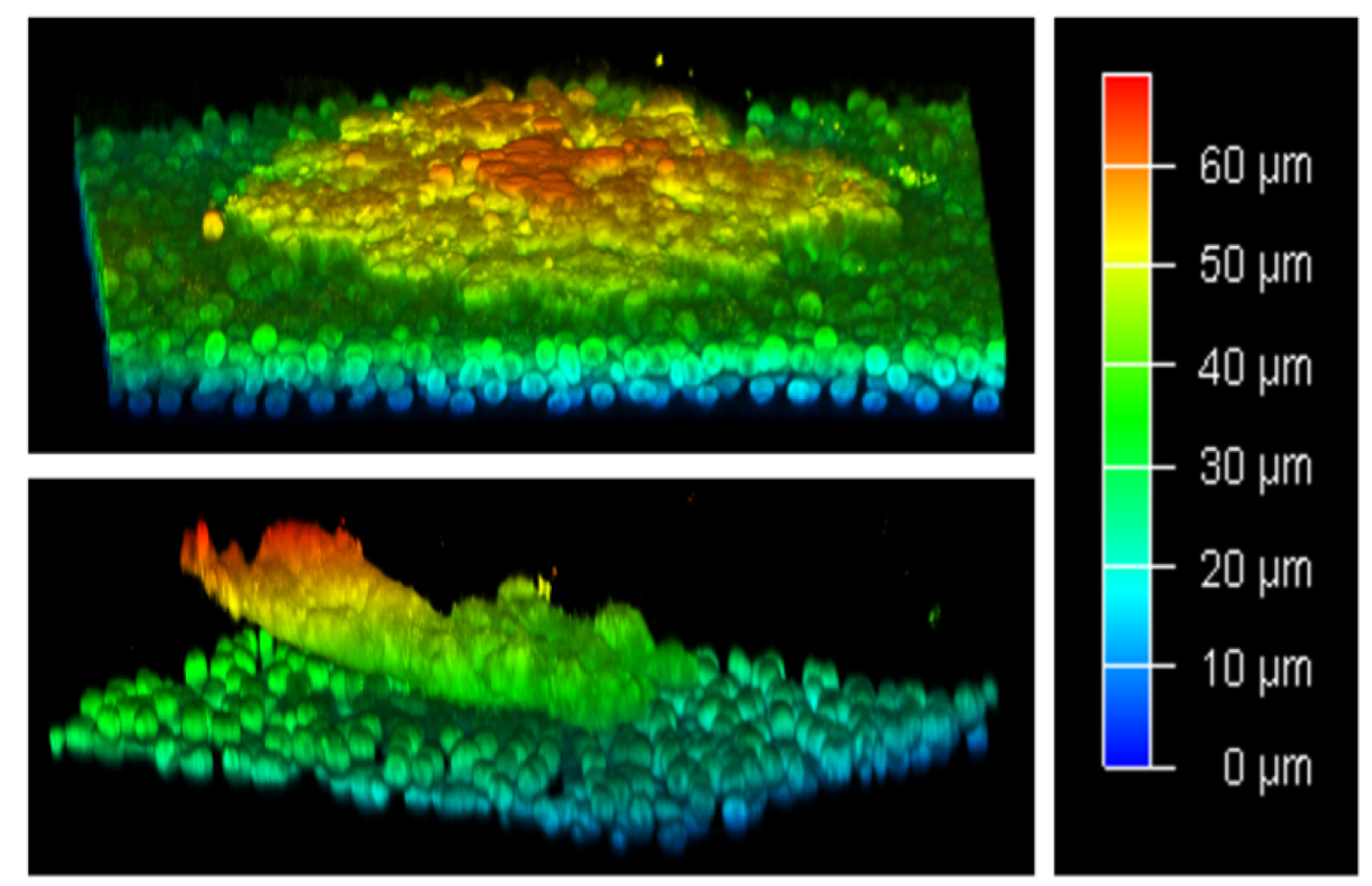

Supplement: S1 Fig — (TIF) [file ppat.1009458.s004.tif]

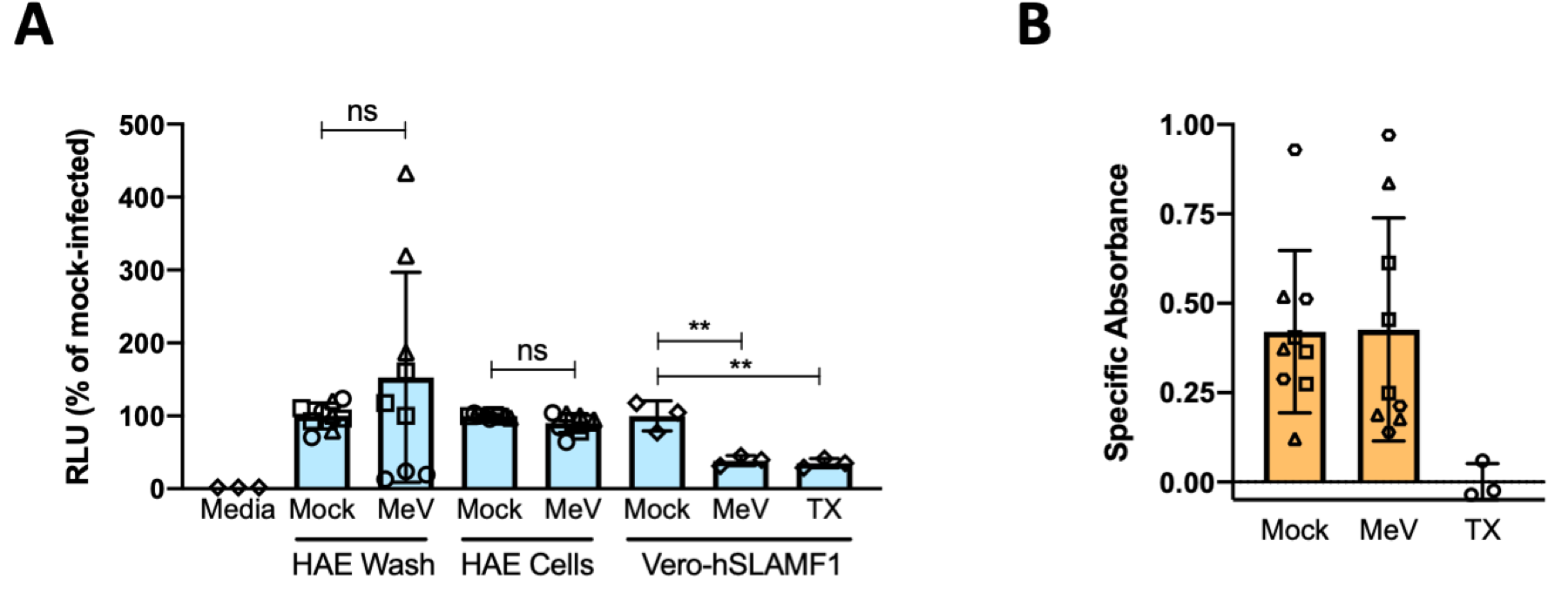

Supplement: S2 Fig — (A) Apical washes were collected from mock or MeV infected HAE at 10 days post-infection. Both cells and washes were lysed and cell viability was measured via a luminescent ATP assay. Vero-hSLAMF1 cells were infected with MeV (MOI = 1) and assayed 48 hours post-infection or treated with 1% Triton X-100 (TX) and assayed 30 minutes later. Luminescence is recorded in relative light units (RLUs) and is reported as a percentage of the matched mock infected condition. Unique shapes indicate unique human donors (n = 3). Means ± standard deviations are shown. Student’s t-tests were performed on raw data values. **p < 0.01 (B) Mock or MeV infected HAE were assayed at 10 days post-infection for cell metabolic functionality via XTT assay. HAE were treated with 1% Triton X-100 for 30 minutes as a control. Formazan conversion was measured via absorbance on a plate reader. Specific absorbance = Absorbance475nm − AbsorbanceBlank − Absorbance660nm. Unique shapes indicate unique human donors (n = 3). Means ± standard deviations are shown. (TIF) [file ppat.1009458.s005.tif]

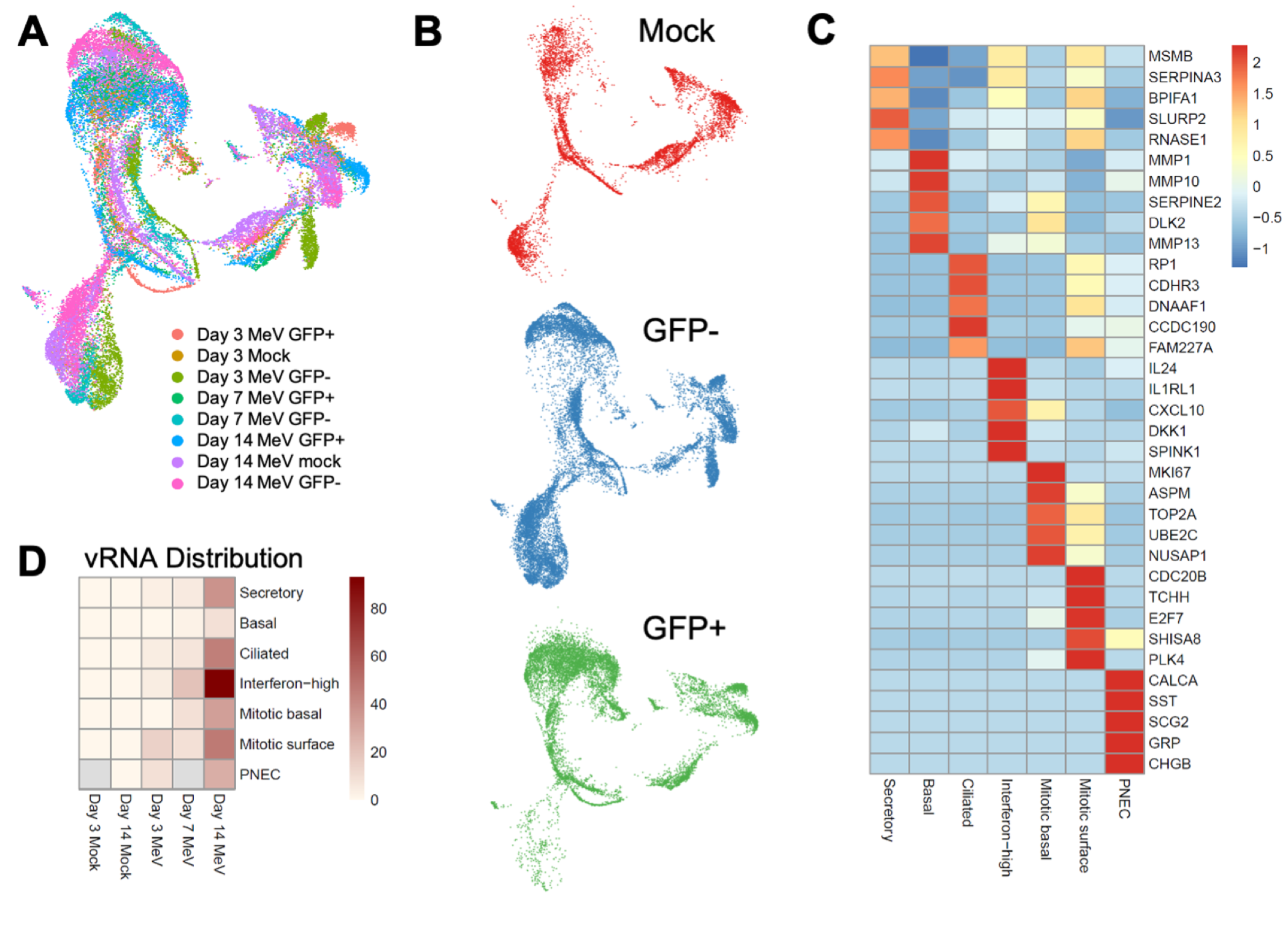

Supplement: S3 Fig — (A) The UMAP projection color-coded by treatment group and timepoint is shown. (B) Individual UMAP projections for each treatment type are shown. (C) The cell marker genes indicated in the heatmap informed cell-type groupings. The color scale corresponds to centered and scaled log(CPM+1). (D) The prevalence of MeV infection across cell types as defined by viral RNA (vRNA) detection is shown. Color intensity corresponds to percentage of cells. (TIF) [file ppat.1009458.s006.tif]

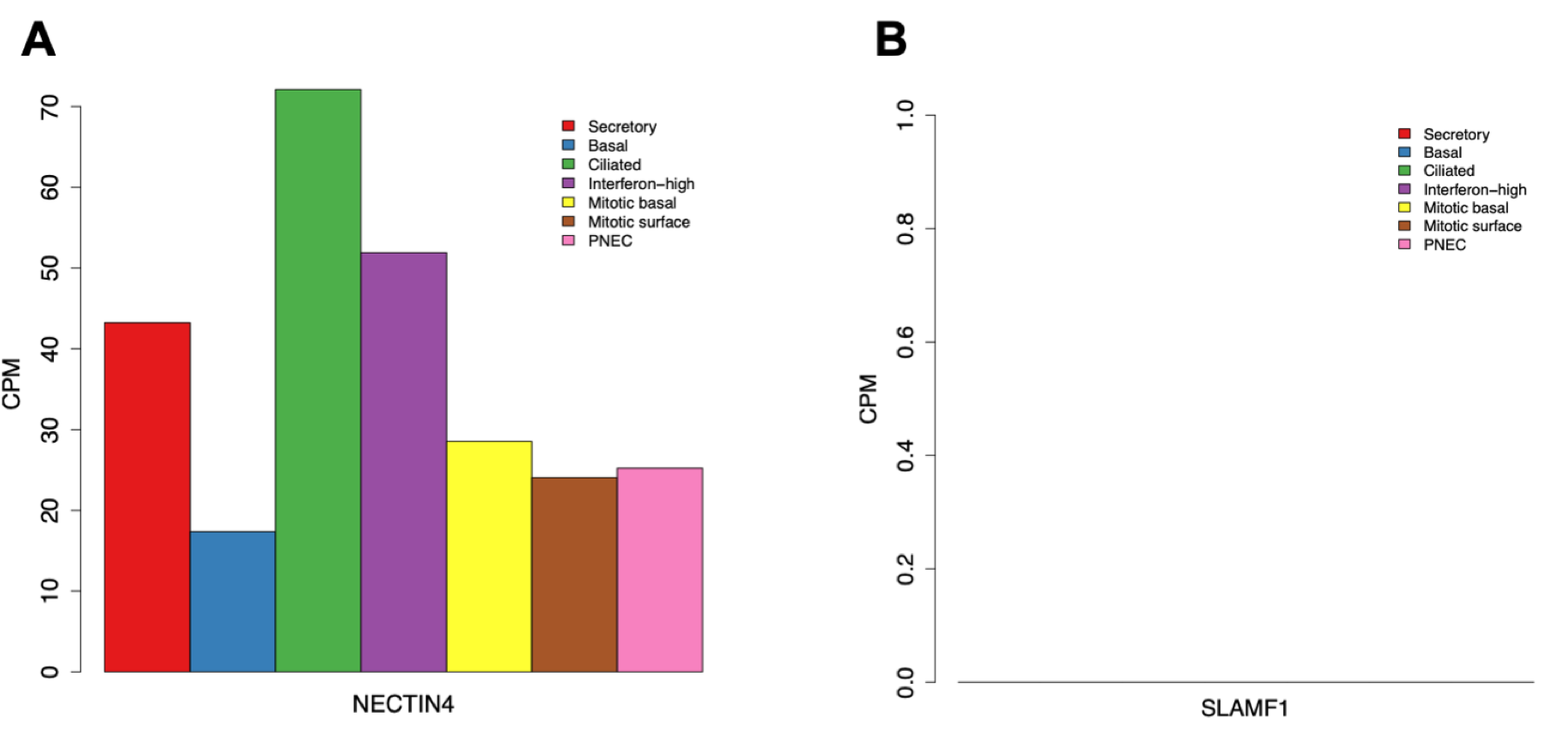

Supplement: S4 Fig — Gene expression in counts per million (CPM) was determined in the scRNA-seq dataset for the two MeV receptors. (A) Nectin-4 was observed in each cell population. (B) SLAMF1 was not detected in any epithelial cell population. (TIF) [file ppat.1009458.s007.tif]

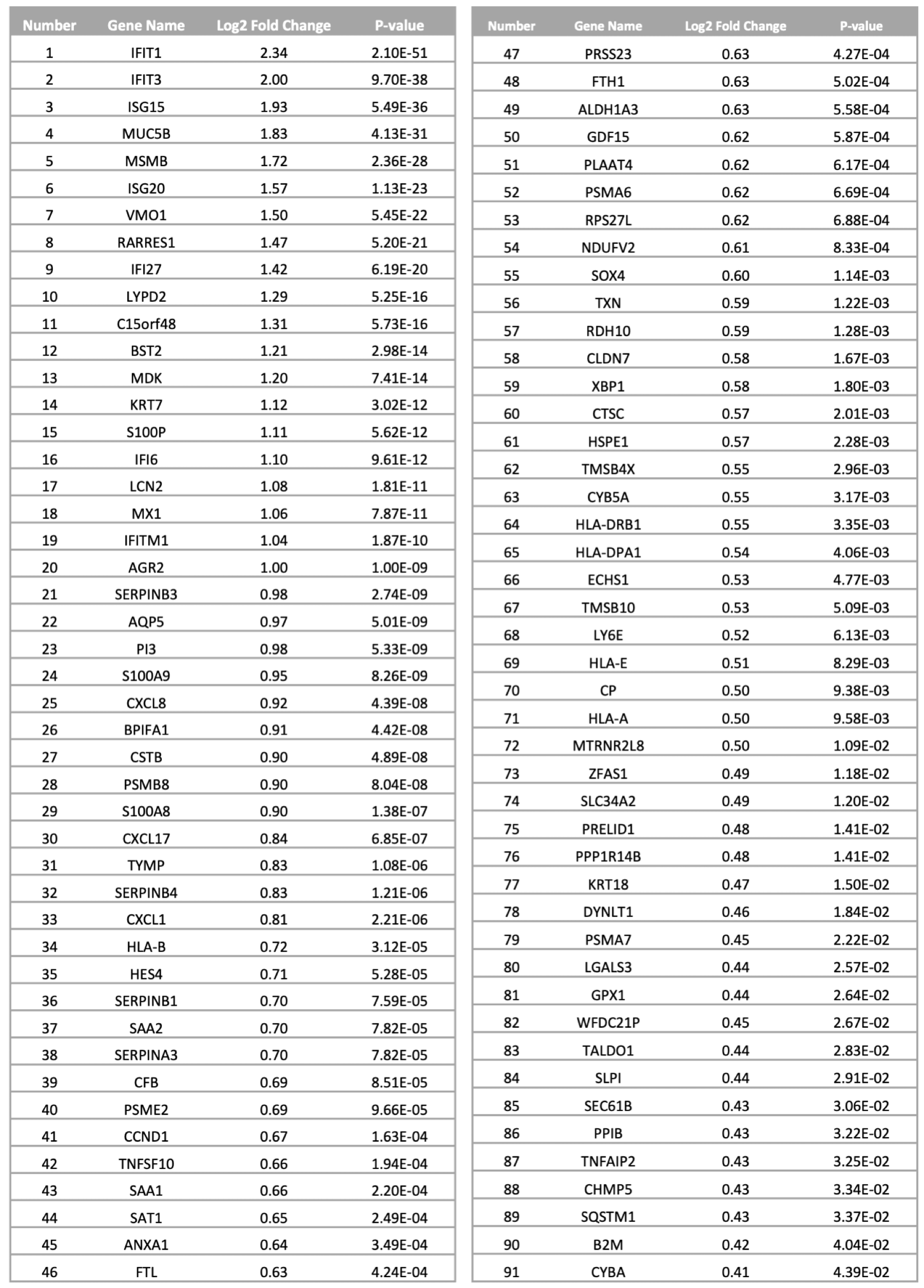

Supplement: S1 Table — (TIF) [file ppat.1009458.s008.tif]

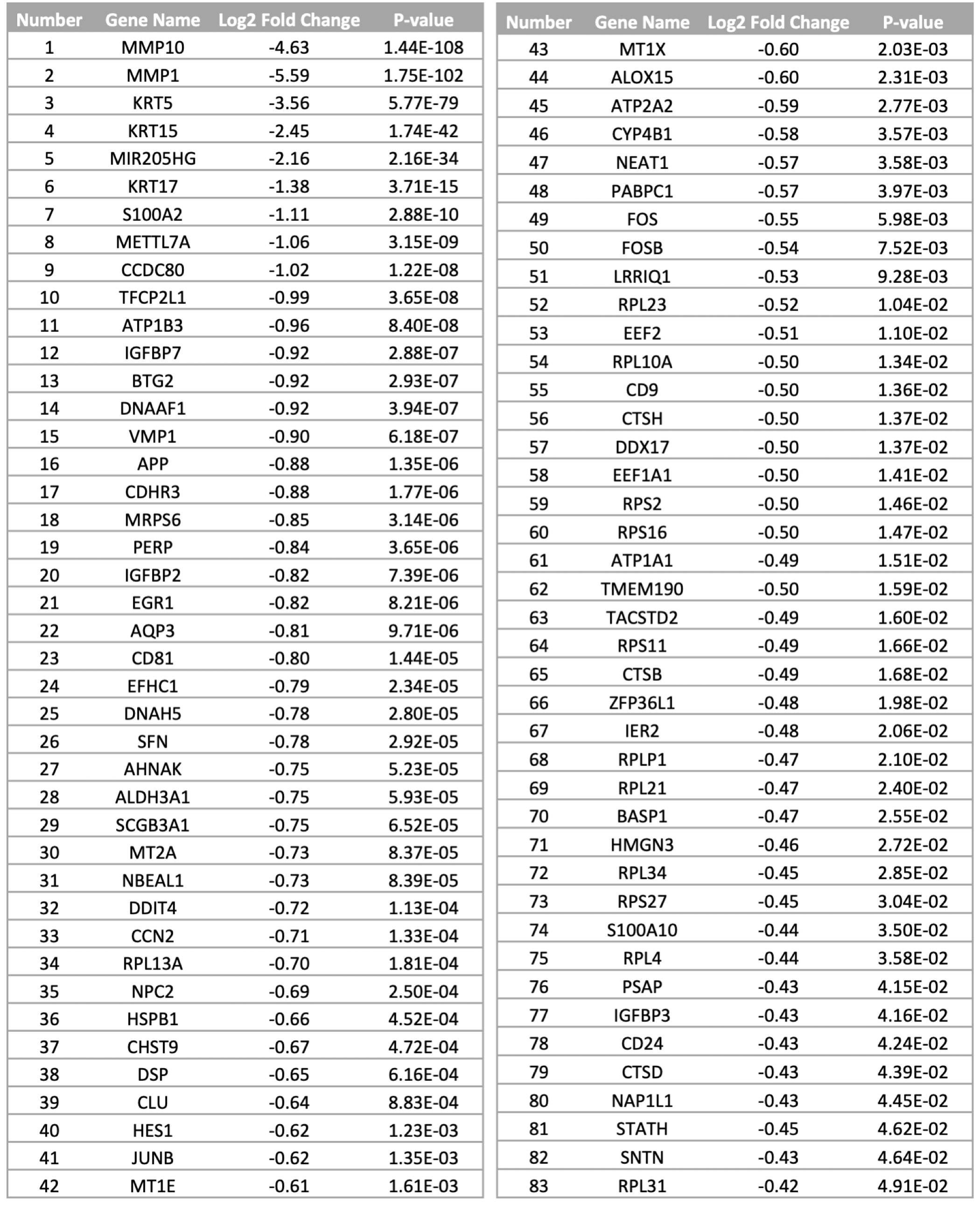

Supplement: S2 Table — (TIF) [file ppat.1009458.s009.tif]

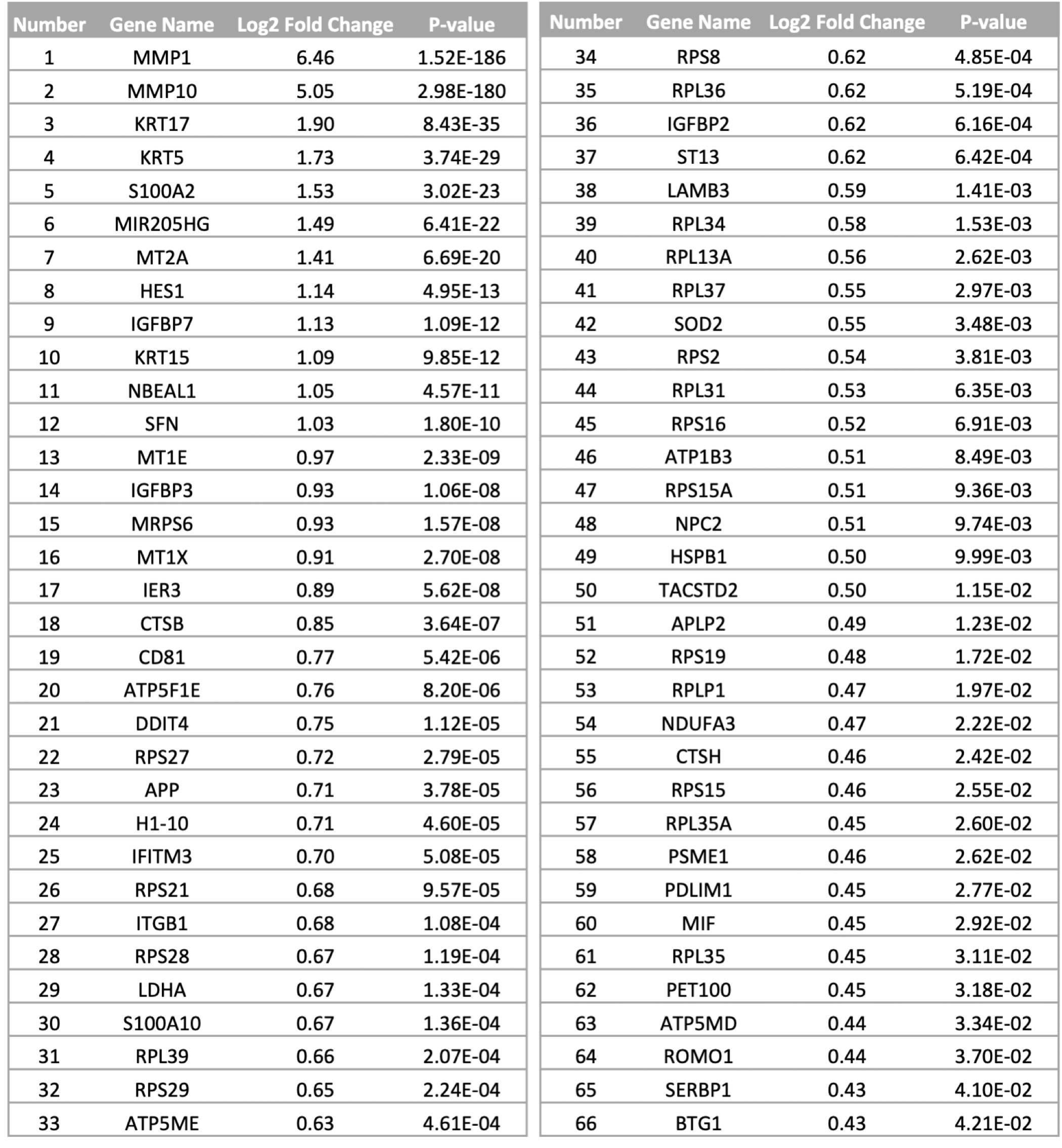

Supplement: S3 Table — (TIF) [file ppat.1009458.s010.tif]

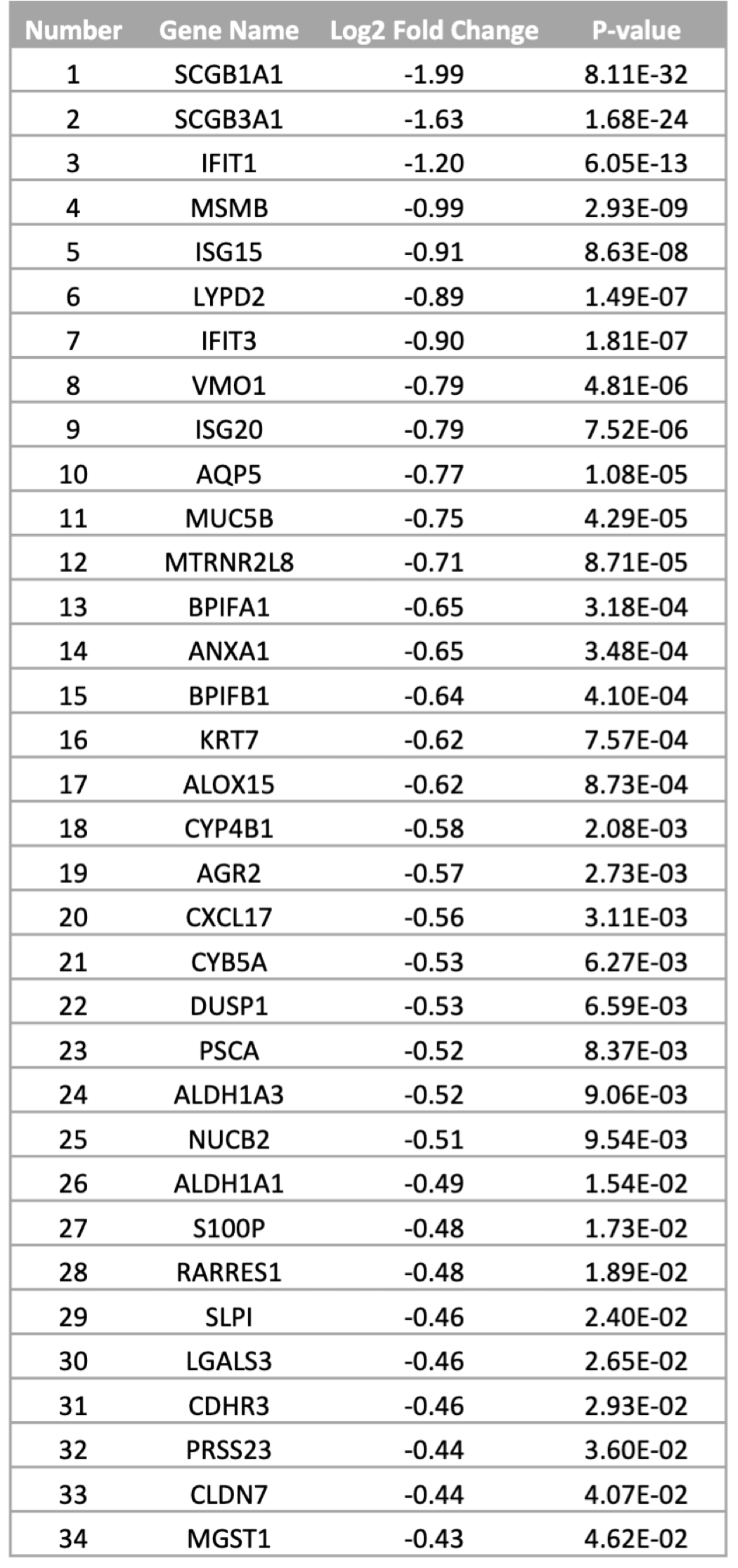

Supplement: S4 Table — (TIF) [file ppat.1009458.s011.tif]
